# Evaluation of olive oil effects on human stress response by measuring cerebral blood flow

**DOI:** 10.1002/fsn3.2099

**Published:** 2021-02-16

**Authors:** Yasue Mitsukura, Brian Sumali, Risa Nara, Kenichi Watanabe, Masami Inoue, Ken Ishida, Mika Nishiwaki, Masaru Mimura

**Affiliations:** ^1^ Faculty of Science and Technology Keio University Kanagawa Japan; ^2^ Department of Neuropsychiatry Keio University School of Medicine Tokyo Japan; ^3^ Graduate School of Science and Technology Keio University Kanagawa Japan; ^4^ J‐OIL MILLS, INC Tokyo Japan

**Keywords:** brain, cerebral blood flow, olive oil, stress, typing task

## Abstract

In this paper, we evaluated the effects of olive oil on human's stress level. In recent years, mental stress from harsh working environment have been causing serious problems to human health, both mentally and physically. Symptoms of stress may include feelings of worthlessness, agitation, anxiety, lethargy, insomnia, and behavioral changes. Additionally, the harsh working environments may cause the workers to adopt unhealthy dietary habits, contributing to the health issue. On the other hand, olive oil has been known to provide stress‐relieving effects both by ingestion and by inhaling the scent. Here, we examined the effects of extravirgin olive oil ingestion for mitigating stress from deskwork. Three best‐selling extravirgin olive oil in Japan were tested, and typing task was selected to emulate deskwork situation. Near‐infrared spectroscopy (NIRS) is utilized in this study to visualize the response in brain via cerebral blood flow analysis and to measure participants’ stress level. Statistical analysis showed that the stress levels were lower during the olive oil ingestion experiment compared to no‐oil experiment, even when measured one hour after the ingestion.

## Graphical abstract

We investigated the effect of oil intake on the brain using cerebral blood flow.

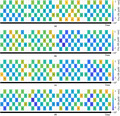

## 1 INTRODUCTION

According to a survey on industrial safety and health in 2018, Japan, approximately 60% of the total workers are faced with work‐related stress (Ministry of Health, Labor and Welfare, 2019). Although a certain level of stress is said to be beneficial for human, continuous long‐term chronic stress adversely affects organisms (Kirby et al., 2013; Leblanc & Vicki, 2009; Schneiderman et al., 2005; Schwabe et al., 2012; Yaribeygi et al., 2017). Stress responses can be categorized into three—physical response, psychological response, and behavioral response, which might occur concurrently. Examples of stress responses include sleeplessness, tiredness, drug dependence, anorexia, and hyperphagia (Etsuo, 2007). In serious cases, chronic stress may lead to life‐threatening diseases such as clinical depression (Constance, 2004). Biologically, when a human experiences “stress,” various changes appear in the body. The levels of cortisol, a steroid hormone, are said to be the biomarker of stress (Atsuo & Kazuhiro, 2004; Coni et al., 2000; Menendez et al., 2005; Perona et al. 2004; Schwingshackl & Hoffmann, 2014; Tuck & Hayball, 2002). An increase in cortisol secretion acts on the immune system, nervous system, and metabolic system, leading to stress reactions such as a decrease in immune function and an increase in heart rate and blood glucose level (Toshihiro et al., 2008). Although salivary cortisol level measurement is the golden standard of objective stress level measurement, analysis using heart rate variability features, standardized questionnaires, and physiological signals have been proposed (Cohen et al., 1983; Crosswell & Lockwood, 2020; Dickerson and Kemeny, 2004; Kim et al., 2018; Nagasawa et al., 2020; Rey et al., 2014; Solomon et al., 1987). One of the physiological signals for measuring stress level was functional near‐infrared spectroscopy (fNIRS). It is one of neuroimaging techniques which works by irradiating the subject with light in the near‐infrared region and examines the change in absorbance of light. It is known that hemoglobin scatters light, and the ratio of infrared light absorbed to that scattered changes depending on the degree of hemoglobin binding with oxygen. NIRS measures this rate of change and the change in oxygenated hemoglobin concentration. As described in conventional researches (Kaga & Kato, 2019; Yoshikazu et al., 2008), fNIRS has been proven as a reliable instrument for qualitative stress level measurement. However, the physiological aspects of the stress response are still largely unknown.

Previous studies on cortisol secretion have shown that lipid intake may suppress the secretion of the hormone (Lee et al., 2015; McEwen, 2008; Ranabir & Reetu, 2011; Toshihiro et al., 2008), and one of the lipid groups that is said to be particularly beneficial to human health were olive oils (Atsuo & Kazuhiro, 2004; Coni et al., 2000; Menendez et al., 2005; Menendez et al., 2005; Schwingshackl & Hoffmann, 2014; Tuck & Hayball, 2002). Although it has been confirmed that olive oil helps to reduce human stress response, the degree of stress mitigation and the reason of olive oil's effectiveness on reducing stress is still not well defined. Additionally, it is also unconfirmed whether all types of olive oils are beneficial or only certain types of olive oils are useful for stress relief. Thus, in this paper, we focused on the effects of olive oil ingestion against mental stress induced by typing task, which measurements were conducted multiple times—directly after ingestion, one hour after ingestion, and two hours after ingestion.

## 2 MATERIALS AND METHODS

### 2.1 Subject demographics

This research was approved by Keio University Bioethics Committee with approval no. 31–66. Originally, 20 healthy university students voluntarily participated in this study, but only 17 of them performed the complete experimental procedures (14 males, 3 females). Written informed consent was obtained from the study participants, including consent to participate and to publish the findings. The subject demographics is available on Table 1.

**TABLE 1:** Experimental conditions. Condition 1 is the control, where the subjects did not ingest any olive oil. On conditions 2, 3, and 4, the subjects ingested 15 g of olive oil along with bread

Condition	1	2	3	4
Intake material	Bread	Bread + Oil A	Bread + Oil B	Bread + Oil C

### 2.2 Experimental procedure

**FIGURE 1:**
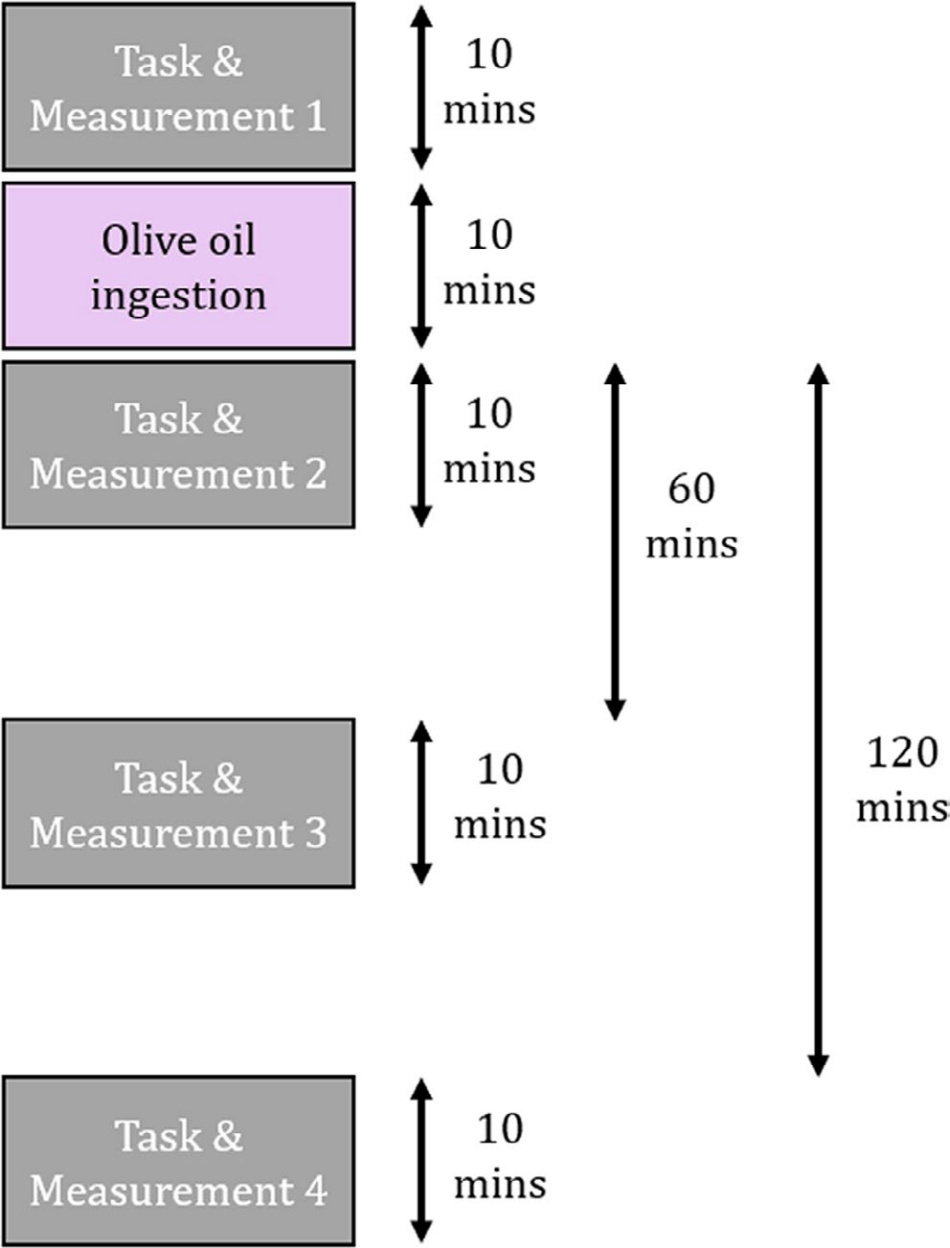
The flow of the experiment

**FIGURE 2:**
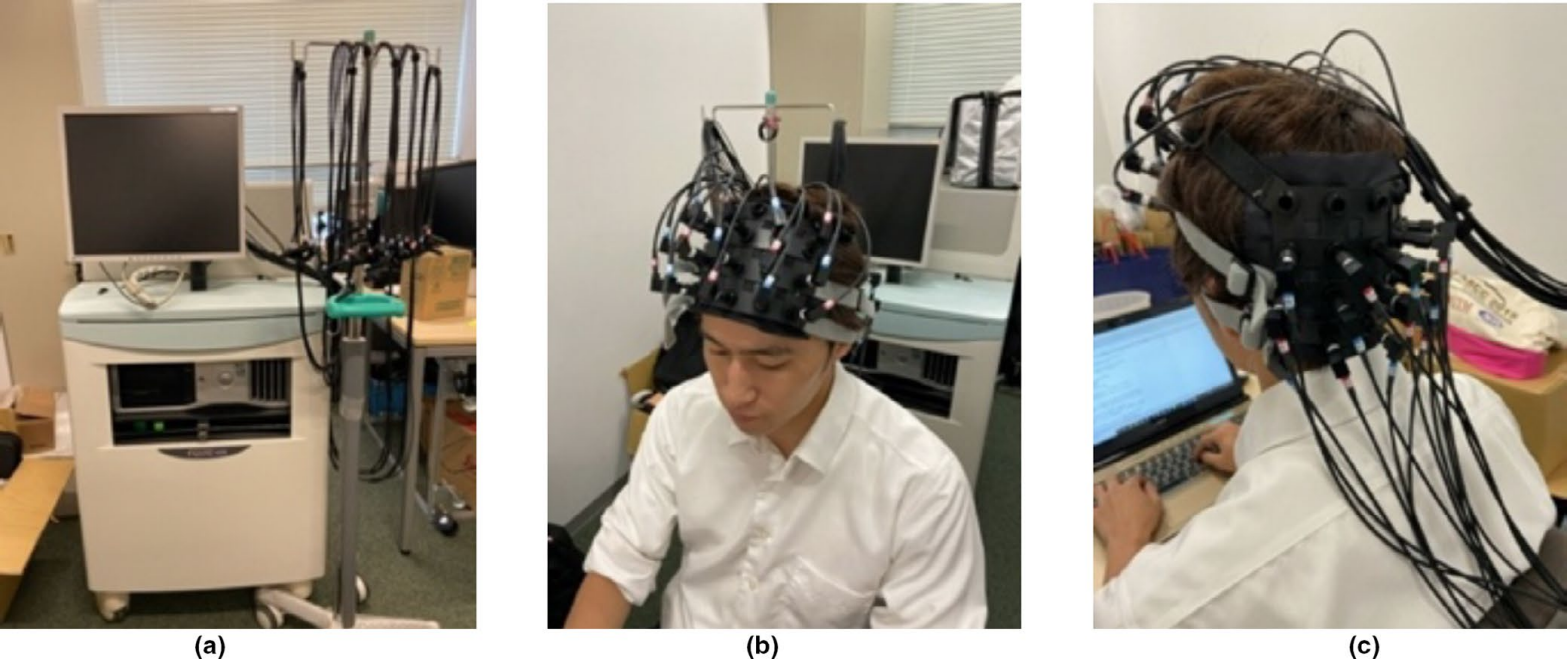
The blood flow extraction device (FOIRE‐3000). (a) (SHIMADZU Co). The data were recorded at 1–22 ch (frontal) and 23–44 ch (occipital), at a sampling frequency of 7.7 Hz (Figure 2 (b), (c) and Figure 3) (Maoka et al., 2016)

The experimental flow is shown on Figure 1, and the photograph of the experimental procedure is shown on Figure 2. The experiment begins with the typing task and NIRS recording session. Then, the participant is instructed to consume the bread with olive oil. Second typing task and NIRS recording begins at 10 min after ingestion (m + 10). Third and fourth tasks start at 60 min after ingestion and 120 min after ingestion, respectively. Four experiments were performed, differing in the consumption contents: Bread‐only as control, bread + olive oil A, bread + olive oil B, and bread + olive oil C. 15 g of olive oil (approx. 1 tbsp) was ingested by the participant during each experiment.

For each experiment, the cerebral blood flow recordings were during typing tasks, and such tasks occurred four times: 10 min before oil ingestion, 10 min after oil ingestion, 60 min after oil ingestion, and 120 min after oil ingestion. The length of typing task is 10 min, and a new research paper is supplied for each session. If the participant completed their assigned task before the time limit, they were instructed to continue the typing task using the next paper. During task session, NIRS signal is recorded.

### 2.3 Materials

Consumption: Bite‐sized white bread and extravirgin olive oils from three different brands were utilized in this study. The three brands were selected from the best‐selling olive oil in Japan, labeled as “Olive oil A,” “Olive oil B,” and “Olive oil C.”

**FIGURE 3:**
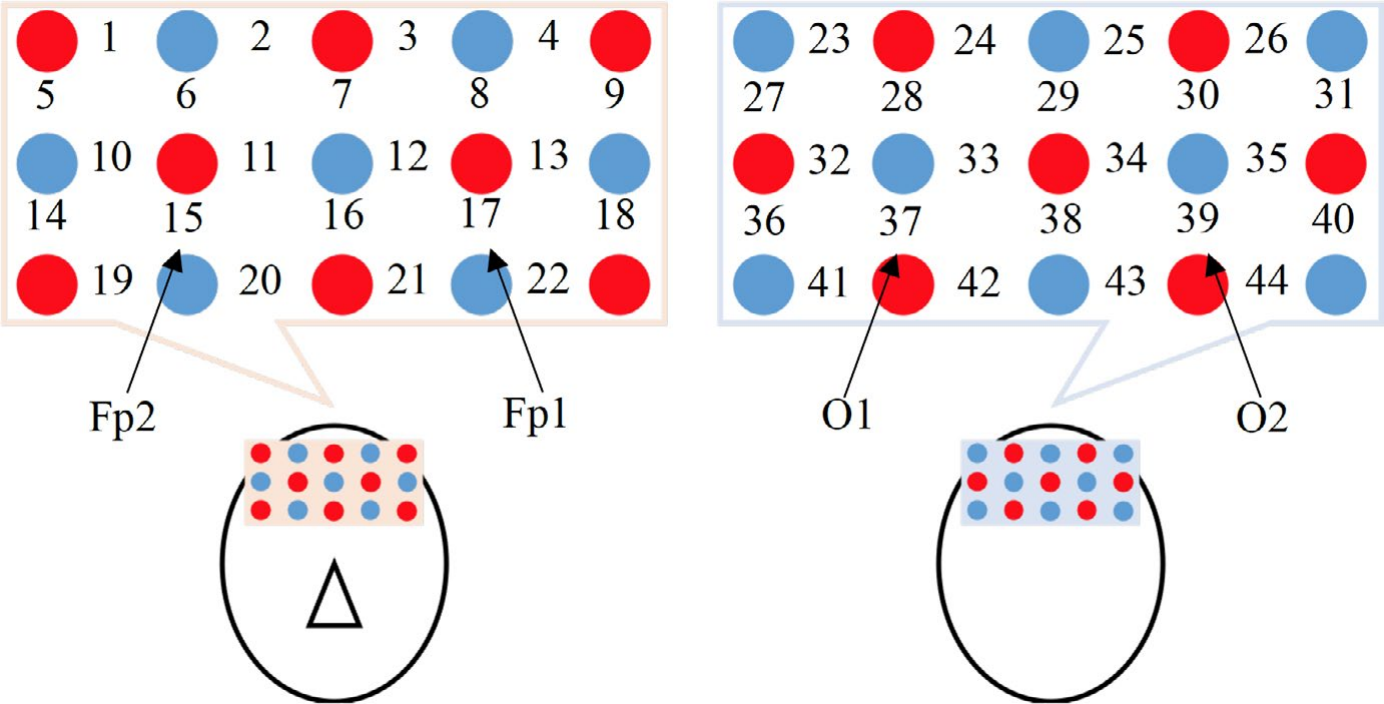
Light emitters and photodetectors are marked by red and blue circles, and the measurement points are indicated by numbers. For frontal, 15 ch and 17 ch were set to Fp2 and Fp1, respectively, according to the international 10–20 system for assuring minimum between subject position variability (Yasue, 2016)

**FIGURE 4:**
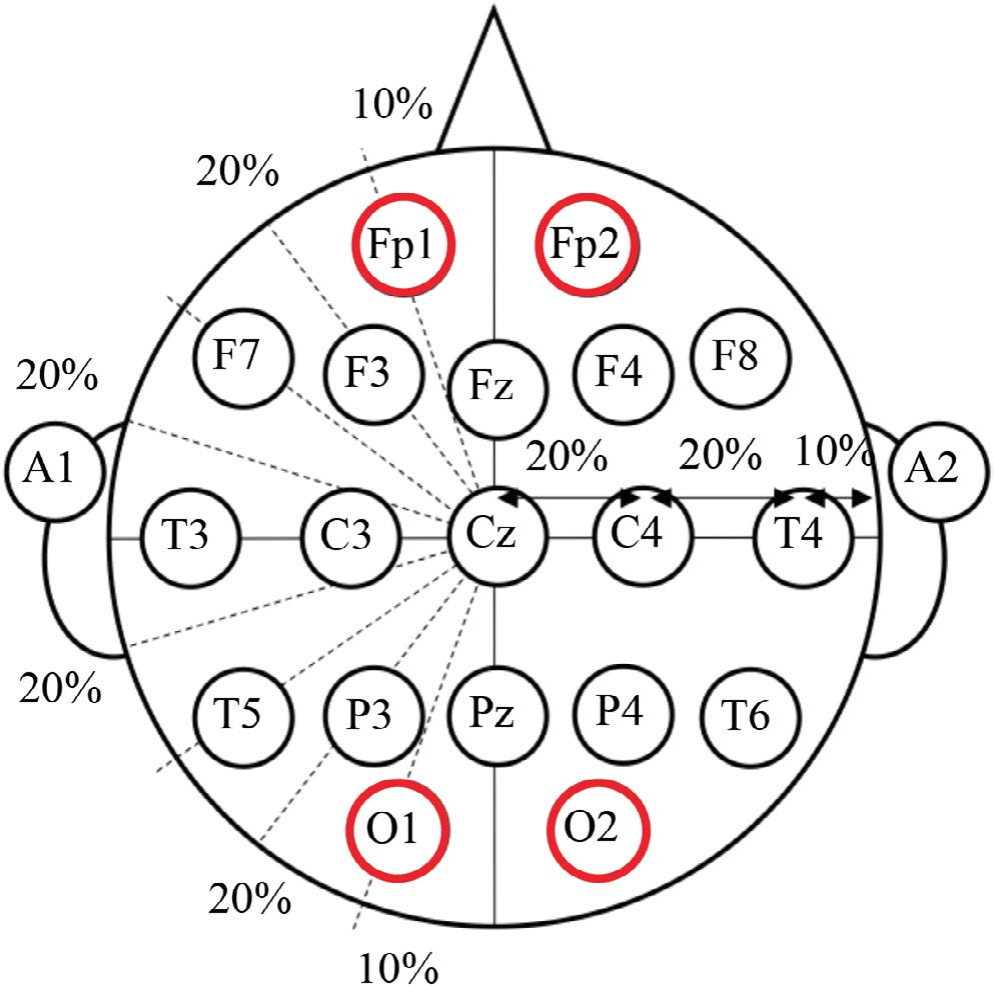
The international 10–20 system

Measurement: FOIRE‐3000 from SHIMADZU Co. for NIRS measurement. The probes for NIRS were placed in the frontal area of the head (channel 1–22) and occipital area (channel 23–44), at a sampling frequency of 7.7 Hz, as shown in Figures 2 and 3. In Figure 3, emitters and detectors are marked by red and blue circles, respectively. Channel 15 and 17 were set to the subjects’ Fp2 and Fp1 location, respectively, and channel 37 and 39 were set to O1 and O2, respectively, according to the international 10–20 system as shown in Figure 4.

Task: A typical typing exercise program “C‐Type” (Sawa, 2002) was utilized for the typing task. Twelve English research papers from publisher Nature were selected as the contents of the typing task (Ben et al., 2019; Sanne et al., 2019; Stephen et al., 2019; Corentin et al., 2019; Philippe et al., 2019; Chris et al., 2019; Elisabeth et al., 2019; Feldmann et al., 2019; Jonathan et al., 2019; Oleg et al., 2019; Hare et al., 2019; Milan et al., 2019). The order of the typing task content was not randomized and is similar for all subjects.

### 2.4 Analysis

To evaluate the effects of fats and oils on stress responses, we analyzed the data acquired by measuring cerebral blood flow. In cerebral blood flow measurement, three types of density data can be acquired: oxyhemoglobin, deoxyhemoglobin, and total hemoglobin. Total hemoglobin is the sum of oxy‐ and deoxyhemoglobin. Herein, the data of oxyhemoglobin were selected for analysis because the changes are the most prominent on oxyhemoglobin. The procedure of our research analysis is as follows:

Step 1: Filtering.

Step 2: Extracting functional component.

Step 3: Standardization.

Step 4: Baseline correction.

Step 5: Calculating integrated values.

First, to remove noise, a low‐pass filter with a cutoff frequency of 1 Hz was applied. The data contain a functional component based on cerebral function and systemic component based on changing posture. Thus, we extracted the functional component using the method of separation into functional and systemic components developed by the National Institute of Advanced Industrial Science and Technology (AIST) in Japan (Toru et al., 2012). The principle of the method is as follows.

The data of oxy‐ and deoxyhemoglobin are shown in Eq 1:

(1) $$\left(\begin{array}{c} \Delta \text{HbO}\\ \Delta \text{HbR} \end{array}\right)=\left(\begin{array}{c} \Delta {\text{HbO}}_{F}\\ \Delta {\text{HbR}}_{F} \end{array}\right)+\left(\begin{array}{c} \Delta {\text{HbO}}_{S}\\ \Delta {\text{HbR}}_{S} \end{array}\right).$$

where ∆HbO and ∆HbR are the values of oxy‐ and deoxyhemoglobin, respectively. ∆HbO_F_, ∆HbR_F_, and ∆HbR_S_ denote the values of functional components and systemic components, respectively. Furthermore, we consider the correlation between functional oxy‐ and deoxyhemoglobin, and the correlation between systemic oxy‐ and deoxyhemoglobin. The brain has a property called neurovascular coupling, wherein oxyhemoglobin will increase and deoxyhemoglobin will decrease by adjusting the regional cerebral blood flow during neural activation. Thus, *k*_F_(−1 < *k*_F_ <0) is defined as a proportional constant, and a proportional relationship is realized between functional oxy‐ and deoxyhemoglobin.

The systemic component changes cause dilation of vasculatures. That is, oxy‐ and deoxyhemoglobin will increase in this process. Thus, *k*_s_ (0 < *k*_s_) is defined as a proportional constant, and a proportional relationship is realized between systemic oxy‐ and deoxyhemoglobin. These relationships can be expressed as follows.

(2) $$\left(\begin{array}{c} \Delta {\text{HbR}}_{F}\\ \Delta {\text{HbR}}_{S} \end{array}\right)=\left(\begin{array}{cc} k_{F} & 0\\ 0 & k_{s} \end{array}\right)\left(\begin{array}{c} \Delta {\text{HbO}}_{F}\\ \Delta {\text{HbO}}_{S} \end{array}\right).$$

Consequently, the following relationships are obtained from both equations.

(3) $$\left(\begin{array}{c} \Delta {\text{HbO}}_{F}\\ \Delta {\text{HbR}}_{F} \end{array}\right)=\frac{1}{k_{F}‐k_{S}}\left(\begin{array}{cc} ‐k_{S} & 1\\ ‐k_{F}k_{S} & k_{F} \end{array}\right)\left(\begin{array}{c} \Delta \text{HbO}\\ \Delta \text{HbR} \end{array}\right).$$

(4) $$\left(\begin{array}{c} \Delta {\text{HbO}}_{S}\\ \Delta {\text{HbR}}_{S} \end{array}\right)=\frac{1}{k_{F}‐k_{S}}\left(\begin{array}{cc} k_{F} & ‐1\\ k_{F}k_{S} & ‐k_{S} \end{array}\right)\left(\begin{array}{c} \Delta \text{HbO}\\ \Delta \text{HbR} \end{array}\right).$$

The value of *k*_F_ is empirically set to −0.6, and the same value was adopted herein. On the contrary, the value of *k*_S_ was decided by minimizing the mutual information between the value of *k*_F_. From the above, the independence of each proportionality constant can be assumed and demonstrating that the occurrence of functional and systemic components is based on different principles is possible.

Subsequently, to consider individual differences, standardization (average: 0; variance: 1) was applied to the extracted functional component. In addition, baseline correction was performed to set the starting point of each data to zero. Furthermore, we calculated the integrated value of the acquired oxyhemoglobin data for 10 min. Lastly, we calculated the average of the integral value for each condition.

## 3 RESULTS

**FIGURE 5:**
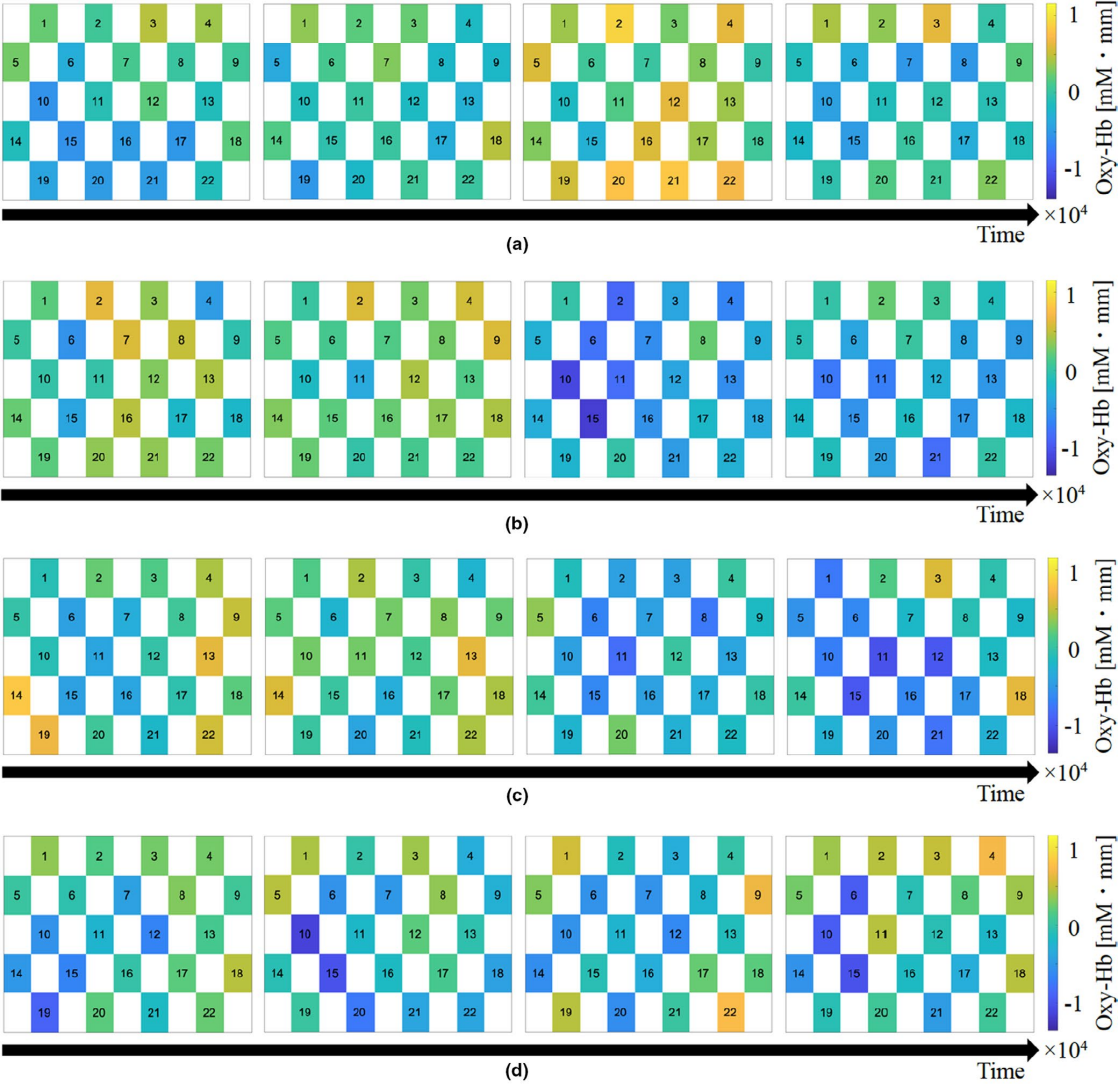
The sample of oxyhemoglobin changes using color maps. The color changes to yellow when oxyhemoglobin increases, and it changes to blue when oxyhemoglobin decreases

**FIGURE 6:**
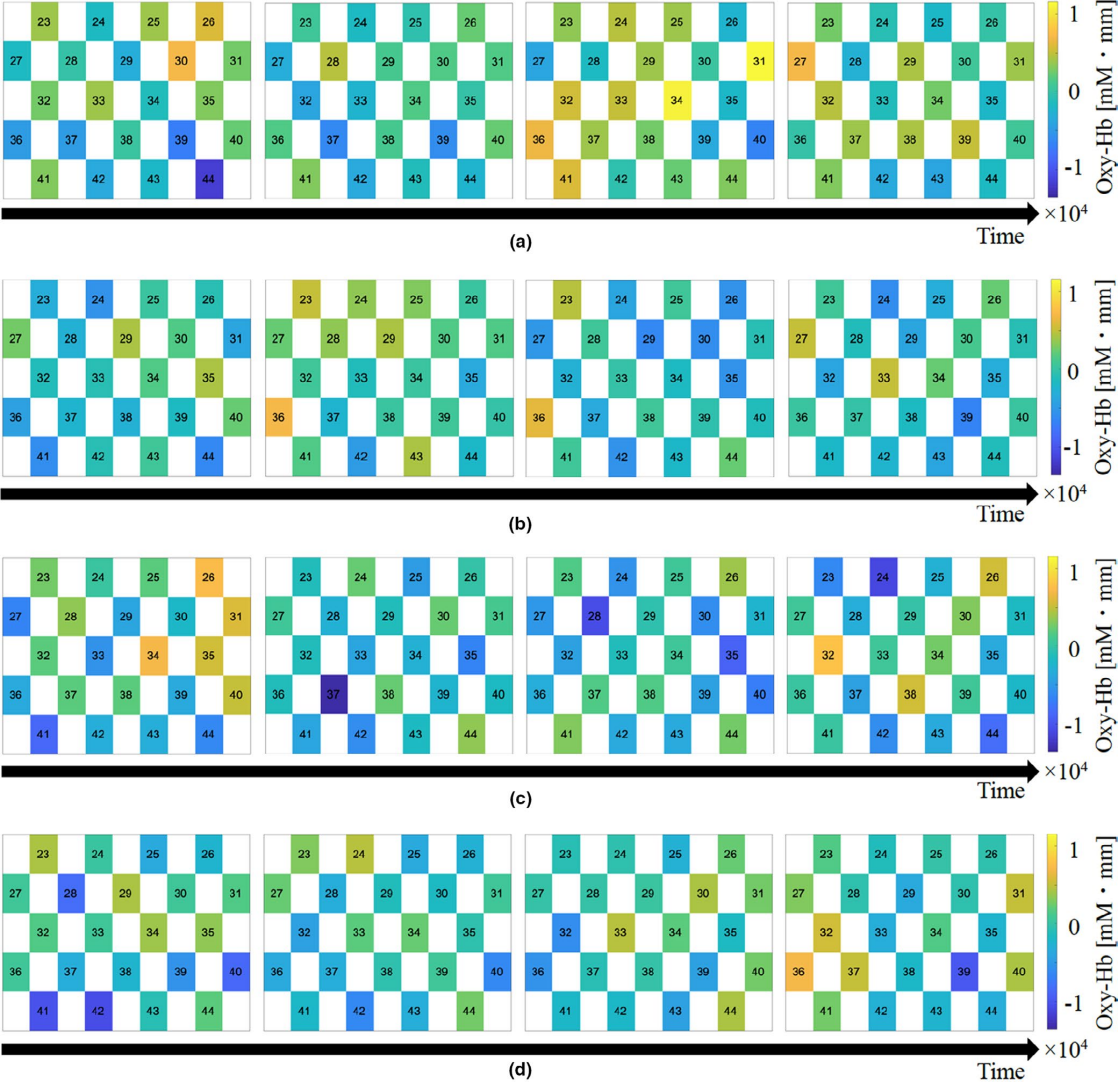
The other sample of oxyhemoglobin changes using color maps

Oxyhemoglobin changes in the frontal and occipital lobes were calculated for each condition. Figures 5 and 6 show oxyhemoglobin changes using color maps. The color changes to yellow when oxyhemoglobin increases, and it changes to blue when oxyhemoglobin decreases.

On comparing the oxyhemoglobin changes in the frontal lobe acquired by four conditions, condition 1 (without intake) tended to increase oxyhemoglobin one hour after bread intake. Besides, conditions 2–4 (with intake) tended to decrease oxyhemoglobin one hour after intake.

Comparing the oxyhemoglobin changes in the occipital lobe acquired by four conditions, condition 1 (without intake) tended to increase slightly when one hour after bread intake. Although the oxyhemoglobin changes in the occipital lobe are less prominent than that of frontal lobe, conditions 2–4 (with intake) tended to decrease oxyhemoglobin one hour after intake.

## 4 DISCUSSION

The limitations of this study are as follows. First, the subjects of this study are only 17 and all of them are Asian in their 20 s. Additionally, we only tested the three olive oils from the best‐selling brands in Japan. Although the usage of olive oil correlates with volume of sales, this does not guarantee that the three olive oils we tested were the most used for direct consumption and not cooking. The task utilized for simulating deskwork was typing test. It was meant to simulate a mundane, repetitious activity, and induce stress. However, it must be acknowledged that job stress is multifaceted in nature and task‐induced stress is only one aspect of it.

As shown in the results section, the main findings of this study are as follows:

- Compared to the control group, consumption of any olive oil reduces the concentration of oxyhemoglobin in the frontal lobe during the typing task and cerebral blood flow measurement conducted on 1 hr after intake and 2 hr after intake.
- Consumption of olive oil did not change the oxyhemoglobin concentration in the occipital lobe.

Currently, a correlation between stress and increased total hemoglobin count has been reported (Yoshikazu et al., 2008; Matthias et al., 2002). Although the subject of analysis of the previous studies is total hemoglobin count and this study only analyzed oxyhemoglobin concentration, the concentration of both total hemoglobin and oxyhemoglobin are said to increase during neural activity due to neurovascular coupling (Peter et al., 1988). In other words, there is a positive correlation between total and oxyhemoglobin. As such, it can be considered that consumption of olive oil reduces the subjects’ stress levels or mitigates the subjects’ stress responses. These results are also in line with the previous study suggesting the trend of stress reduction using electrocardiogram (Atsuo & Kazuhiro, 2004). Additionally, a previous study found correlation between stress and prefrontal brain activity (Richard & William, 1999). This is also in line with our results that oxyhemoglobin concentration was decreased only in the frontal area and not in the occipital area.

We also found that while there is a decrease in oxyhemoglobin concentration at the frontal lobe, during the tasks performed 1 hr after consumption and 2 hr after consumption, there were no changes in oxyhemoglobin concentration during the task performed right after the consumption. This suggests that while olive oil ingestion is beneficial, the stress mitigation effect is not instantaneous. The time delay might also be related to the fact that the digestion and absorption of fats and oils are around two to three hours.

## 5 CONCLUSION AND FUTURE WORK

We recorded the cerebral blood flow activity of 17 healthy university students during typing tasks. As control, the participants were instructed to consume a piece of bread and as the experiment, the participants were instructed to consume a piece of bread along with 15 g of extravirgin olive oil. The measurement was conducted four times, 10 min before the ingestion, right after the ingestion, 1 hr after ingestion, and 2 hr after ingestion. Analysis on oxyhemoglobin concentration found that compared to the control, ingestion of 15 g extravirgin olive oil of any brand reduces the oxyhemoglobin concentration during the measurements conducted on 1 hr after ingestion and 2 hr after ingestion.

Our work is by no means complete. We need to address the limitations we specified before. 17 subjects with average age of 20 s certainly do not completely reflect the workforce population, and more samples with average age and sex ratio corresponding to the workforce population are needed. Second, we regarding the olive oils. We utilized the three best‐selling extravirgin olive oils in Japan, and all of them produced similar result. Rather than a brands of olive oil, focus on the chemical contents might reveal more information. Finally, more types of stress stimulation tasks might be considered.

## CONFLICT OF INTEREST

The authors declare no conflict of interest.

## **AUTHOR** **CONTRIBUTIONS**

YM, KW, MI, KI, MN, and MM conceptualized the data. YM, RN, and BS involved in data curation, formal analysis, investigation, and writing—original draft. YM involved in funding. YM, RN, BS, KW, MI, KI, and MN involved in methodology. YM and MM involved in project administration. YM, KW, MI, KI, and MN involved in resources. RN and BS involved in software. YM and MM involved in supervision. YM, BS, and MM involved in validation. RN and BS involved in visualization. YM and BS involved in writing—review and editing.

6

**TABLE 2:** Subject demographics. *N* = 17

Asian (%)	100
Female (%)	17.6
Age	22.8 ± 1.3 years

**TABLE 3:** Fatty acid composition of each olive oil

Fatty acid composition (%)		Oil A	Oil B	Oil C
C16:0	Palmitic acid	12.6	9.9	12.5
C18:0	Stearic acid	2.0	2.7	2.5
C18:1	Oleic acid	72.7	79.4	73.1
C18:2	Linoleic acid	9.9	5.3	8.7
C18:3	a‐Linolenic acid	0.7	0.6	0.7
others		2.1	2.0	2.4

## Data Availability

Research data are not shared.
